# A direct assessment of human prion adhered to steel wire using real-time quaking-induced conversion

**DOI:** 10.1038/srep24993

**Published:** 2016-04-26

**Authors:** Tsuyoshi Mori, Ryuichiro Atarashi, Kana Furukawa, Hanae Takatsuki, Katsuya Satoh, Kazunori Sano, Takehiro Nakagaki, Daisuke Ishibashi, Kazuko Ichimiya, Masahisa Hamada, Takehisa Nakayama, Noriyuki Nishida

**Affiliations:** 1 Department of Molecular Microbiology and Immunology, Nagasaki University Graduate School of Biomedical Sciences, 1-12-4 Sakamoto, Nagasaki 852-8523, Japan; 2 Department of Locomotive Rehabilitation Science, Nagasaki University Graduate School of Biomedical Sciences, 1-7-1 Sakamoto, Nagasaki 852-8523, Japan; 3 Department of Physiology and Pharmacology, Faculty of Pharmaceutical Sciences, Fukuoka University, 8-19-1 Nanakuma, Jonan-ku, Fukuoka 814-0180, Japan; 4 Kripton Co., Ltd., Dai 12 Daitetsu Bldg. 7F., 4-3-12 Yotsuya, Shinjuku-ku, Tokyo 160-0004, Japan

## Abstract

Accidental transmission of prions during neurosurgery has been reported as a consequence of re-using contaminated surgical instruments. Several decontamination methods have been studied using the 263K-hamster prion; however, no studies have directly evaluated human prions. A newly developed *in vitro* amplification system, designated real-time quaking-induced conversion (RT-QuIC), has allowed the activity of abnormal prion proteins to be assessed within a few days. RT-QuIC using human recombinant prion protein (PrP) showed high sensitivity for prions as the detection limit of our assay was estimated as 0.12 fg of active prions. We applied this method to detect human prion activity on stainless steel wire. When we put wires contaminated with human Creutzfeldt–Jakob disease brain tissue directly into the test tube, typical PrP-amyloid formation was observed within 48 hours, and we could detect the activity of prions at 50% seeding dose on the wire from 10^2.8^ to 10^5.8^ SD_50_. Using this method, we also confirmed that the seeding activities on the wire were removed following treatment with NaOH. As seeding activity closely correlated with the infectivity of prions using the bioassay, this wire-QuIC assay will be useful for the direct evaluation of decontamination methods for human prions.

Prion diseases, also known as transmissible spongiform encephalopathies (TSEs), such as bovine spongiform encephalopathy in cattle, scrapie in sheep and Creutzfeldt–Jakob disease (CJD) in humans, are fatal neurodegenerative disorders. At present, there is no effective therapy available for the diseases^1^. A host encoded normal prion protein, PrP^C ^2, is required for susceptibility to prion infection^3^,^4^,^5^, and a hallmark of prion diseases is the accumulation of misfolded forms of PrP, PrP^Sc^. This amyloidogenic abnormally folded protein can be infectious. In human prion diseases, most cases (80%) are categorised as sporadic and approximately 15% of cases are a genetic form carrying a mutation in the prion protein gene *PRNP*. Less than 1% are caused by accidental transmission^6^. The possible iatrogenic transmission of such diseases was originally pointed out by Gajdusek in 1970s when the transmissibility of Kuru and CJD was evidenced^7^. In Japan, more than 140 cases of iatrogenic CJD have been identified following dura mater grafting from 1985 until now^8^. Accidental iatrogenic transmission of sporadic CJD (sCJD) has only occurred during neurosurgical procedures^9^. Until now, no cases of iatrogenic transmission following general surgery of nervous tissue or through endoscopic procedures have been reported^9^. Furthermore, experimental transmission studies using non-human primates demonstrated that bodily secretions are not infectious and that potential prion contamination of endoscopic instruments is not sufficient to cause human-to-human transmission^10^. In the case of CJD, infectivity is limited to the central nervous system; however, more recently many peripheral tissues from patients with valiant CJD have been shown to be infectious, and PrP^Sc^ has also been detected in lymphoid organs such as the thymus, tonsils and spleen^11^. Moreover, PrP^Sc^ has been detected in muscle, but no evidence of iatrogenic transmission was reported, suggesting a risk of iatrogenic transmission via contaminated surgical instruments^12^.

Infectious agents are highly resistant to routine decontamination methods^13^. High concentrations of sodium hydroxide, sodium hypochlorite or prolonged steam sterilisation are recommended methods for prion disinfection; however, most methods damage the surgical instruments^14^,^15^,^16^,^17^. Therefore, the development of new disinfection methods is needed for the safe handling and reprocessing of surgical instruments. To estimate the effectiveness of the methods, the evaluation of prion activity is of key importance.

Because of the lack of nucleic acid components, approaches for TSE rely upon methods of immunodetection including immunohistochemistry and enzyme-linked immunosorbent assay using antibodies against PrP^18^,^19^,^20^,^21^. Another evaluation method is Western blotting for protease-resistant PrP^22^. However, the detection range of Western blotting is narrow and not suitable to evaluate the decontamination of prion seeds. For evaluation of prion decontamination, the prion contaminated stainless steel wire test has often been used and infectivity assessed using a bioassay^23^,^24^,^25^,^26^. However, bioassays are needed for at least 1 year to quantify the infectivity, even if transgenic mice expressing PrP are used^27^. Recently, various *in vitro* PrP^Sc^ formation methods were developed. We have shown that a new *in vitro* amplification technology called real-time quaking-induced conversion (RT-QuIC) is highly sensitive for human prion and useful for detecting small amounts of PrP^Sc^ in cerebrospinal fluid. For the RT-QuIC reaction, intermittent shaking enhances the conversion of soluble recombinant PrP into amyloid fibrils only in the presence of PrP^Sc ^28,29,30.

Here, we show that a new modified method named wire-QuIC can be applied for the direct evaluation of prion activity. Prion seeds 263K and sCJDs could firmly bind to stainless steel wire and gave rise to QuIC-positive reactions. Moreover, we demonstrated that treatment of wire with 1 mol/L NaOH solution was suitable for decontamination of prions. These results indicate that wire-QuIC can be useful to evaluate the decontamination of human prions on medical devices such as surgical instruments.

## Results

**Figure 1:**
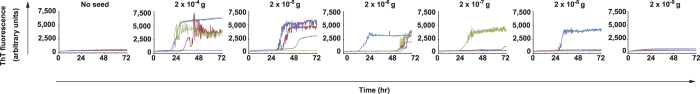
Stainless steel-wire with small amounts of prion seed attached can be detected by RT-QuIC reaction (wire-QuIC). The limiting dilution of 263K-BH was used for wire-QuIC reaction. Fibril formations of recombinant PrP were visualised by measurement of ThT fluorescence.

To determine whether stainless steel wire is viable and does not affect amyloid formation, we conducted RT-QuIC using prion-seed-contaminated wire (wire-QuIC) instead of liquid brain homogenates (BH). To compare the efficiency of wire-QuIC, the classical RT-QuIC reaction with liquid BH was performed in parallel. As shown in Supplementary Fig. S1, the RT-QuIC reaction can detect prion seeding activities in more than 10^−8^ g of BH. Importantly, the QuIC signal could also detect prion seeds attached to the wire (Fig. 1). Wire-QuIC could detect prion seeds in more than 2 × 10^−8^ g of 263K-BH.

**Figure 2:**
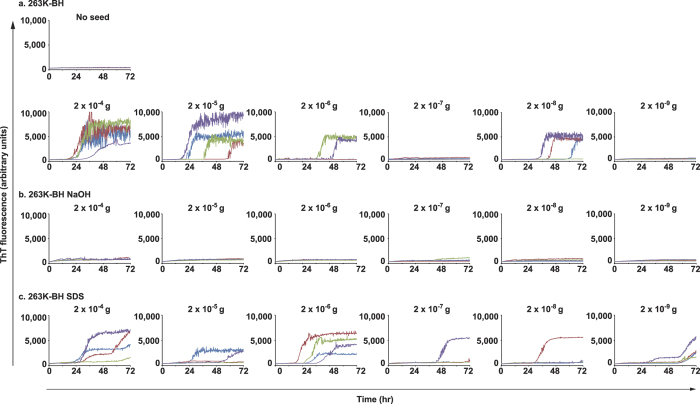
Wire-QuIC reaction can be useful to evaluate the decontamination rate of 263K hamster prions. Decontamination of prion 263K treated with NaOH or SDS was evaluated by wire-QuIC reaction. Dilutions of 263K-BH (10-fold) were attached to wires, and then were treated with 1 mol/L of NaOH for 2 h (b) or 3% (w/v) SDS at 100 °C for 10 min (c). Wire-QuIC reactions were performed to measure the residual prion seeds to evaluate the decontamination rates of prion.

To determine whether the wire-QuIC reaction was useful to evaluate the decontamination rate of prion from instruments such as medical equipment by washing procedures, wires with attached 263K-BH were treated with two prion-inactivation procedures (1 mol/L of NaOH solution for 2 h and 3% (w/v) sodium dodecyl sulfate (SDS) solution at 100 °C for 10 min). The positive signal of 263K-prion-seeds was lost after treatment with 1 mol/L of NaOH solution (Fig. 2b). In contrast, no significant deletion of signal was obtained from wire-QuIC-reaction incubated with SDS solution (Fig. 2c). The same results were obtained using the classical method of RT-QuIC with liquid BHs (Supplementary Fig. S1). Although NaOH treatment removed the positive signal of RT-QuIC, there was no significant change following incubation with SDS solution.

**Figure 3:**
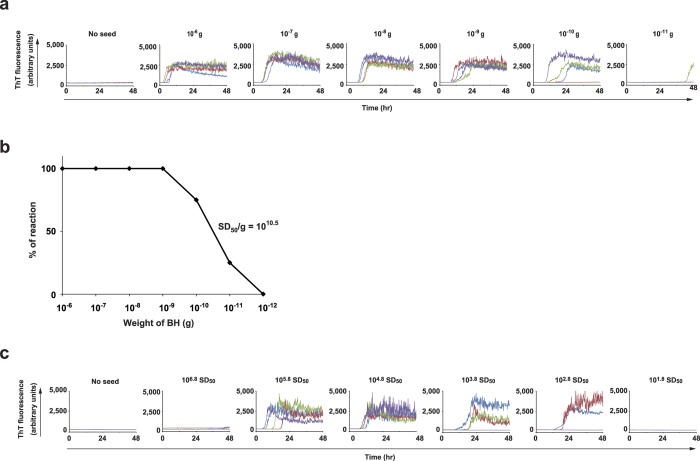
Wire-QuIC reaction can detect human prion seeds from contaminated stainless steel wires. (**a**) Limiting dilution of sCJD-BH was used for RT-QuIC reaction. Fibril formations of recombinant PrP were visualised by measurement of ThT fluorescence. (**b**) Curve of seeding dose activities. Seeding dose 50% (SD_50_) per gram was 10^10.5^. (**c**) Limiting dilution of sCJD-BH was used for wire-QuIC reaction.

To determine whether wire-QuIC reaction can also detect human prion, we used wire-QuIC reaction with sCJD patient BH. The positive signal of sCJD-prion-seeds was detected in more than 10^−11^ g of brain using the classical method of RT-QuIC with liquid BHs (Fig. 3a). The relative concentration of prion-seeding activity, which is the number of seeding doses giving 50% positive replicate reactions (SD_50_) per unit of tissue, as determined by end-point dilution RT-QuIC was 10^10.5^ SD_50_/g brain (Fig. 3b)^28^. In accord with the results of 263K-BHs (Fig. 1), wire-QuIC reaction with sCJD-BH could detect prion seeding activities (Fig. 3c). However, the wire-QuIC reaction had a lower sensitivity than the RT-QuIC reaction, and could detect more than 10^2.8^ SD_50_. Importantly, there was no signal in wire-QuIC with a wire that attached high concentrations (10^6.8^ SD_50_) of sCJD-BH (Fig. 3c).

**Figure 4:**
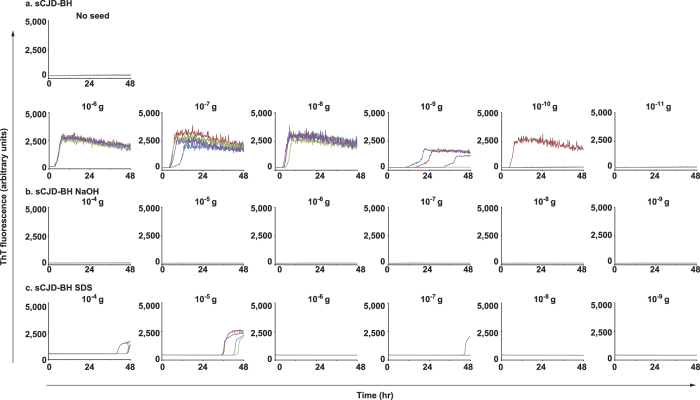
Inactivation of human prion seeds treated with NaOH or SDS solution, as evaluated by RT-QuIC reactions. Dilutions of sCJD-BH (10-fold) were treated with 1 mol/L NaOH solution for 2 h (b) or 3% (w/v) SDS solution at 100 °C for 10 min (c). RT-QuIC reactions were performed to measure the residual prion-seeding activities.

**Figure 5:**
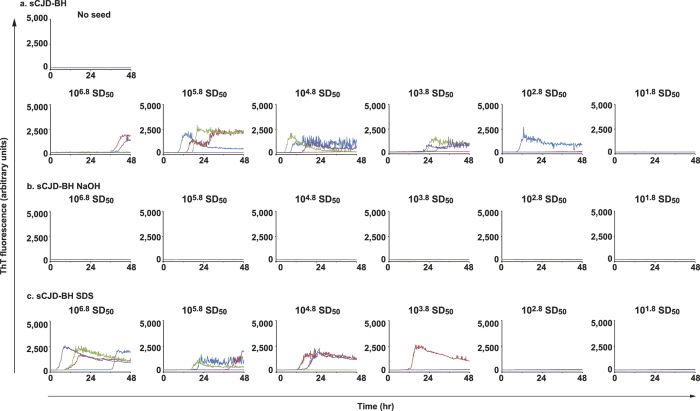
Wire-QuIC reaction can be useful to evaluate the decontamination rate of human prions. Decontamination of human sCJD prion with NaOH or SDS were evaluated by wire-QuIC reactions. Dilutions of sCJD-BH (10-fold) were attached to wires and then were treated with 1 mol/L of NaOH solution for 2 h (b) or 3% (w/v) SDS solution at 100 °C for 10 min (c). Wire-QuIC reactions were performed to measure the residual prion seeds to evaluate the decontamination rates of prion.

For decontamination rate experiments, where sCJD-BH was treated with NaOH or SDS solution, the residuals of prion-seeds were tested by RT-QuIC and wire-QuIC. In accord with results of 263K-BHs, in the RT-QuIC reaction, sCJD prion seeds were inactivated after NaOH treatment (Fig. 4b), but partial inactivation was obtained from treatments with SDS solution (Fig. 4c). The same results were obtained in wire-QuIC experiments (Fig. 5). Importantly, wires contaminated with high concentrations of sCJD-BH (10^6.8^ SD_50_) were QuIC-positive when treated with SDS (Fig. 5c). This phenomenon may be because excessive dirt came off or detergents stabilised the prion-seed structure. These results suggest that the wire-QuIC reaction is useful for evaluating human sCJD prion-seed decontamination.

**Figure 6:**
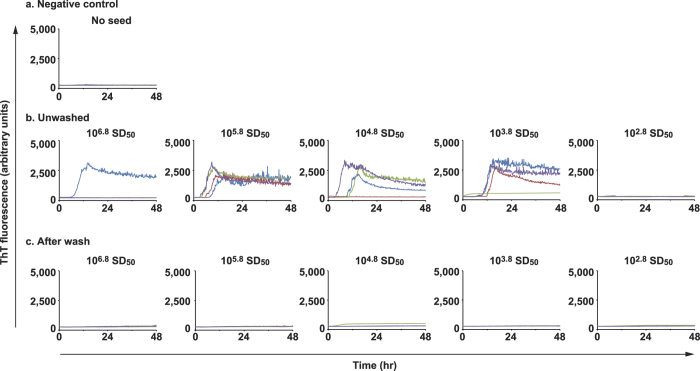
Evaluation of cleaning using the new washing procedure by wire-QuIC reaction. Dilutions of sCJD-BH (10-fold) were attached to wires and then washed using the new procedure described in “Materials and Methods.” Wire-QuIC reactions were performed to measure residual prion seeds.

We developed a new washing procedure for rigid endoscopes for which electrolysis water and sonication are used. To test the decontaminating efficiency of human prion, wires that attached 10-fold diluted sCJD-BH were washed as described in “Materials and Methods” in 1.5 mL tubes, and then evaluated using the wire-QuIC reaction. No positive signal was detected with the wire-QUIC reaction (Fig. 6).

## Discussion

A basic problem to prevent iatrogenic transmission of prion diseases is the lack of a convenient system to detect infectious prion on surgical instruments. In this study, we modified RT-QuIC to evaluate the residual prion seeds on wires (wire-QUIC). Although normal RT-QuIC is more suitable for and more sensitive in detect prion in cerebrospinal fluid or the brain, the wire-QuIC can detect dried prion seeds attached to wire. Both 263K-prion (Fig. 1) and sCJD prion (Fig. 3) on wire was amplified *in vitro.* This finding is in agreement with other studies showing that stainless steel wire can bind prion seeds firmly, and that surface-bound prions can transmit scrapie to recipient mice^23^,^24^. Notably, there was no signal in QuIC with high concentrations of sCJD-BH attached to the wire (Fig. 3c), and no seeding activity was observed at 2 × 10^−7 ^g brain dilution, while it was present at 2 × 10^−8 ^g (Fig. 2a). These paradoxical results may reflect the fact that the QuIC reaction is extremely sensitive and may be influenced by unknown inhibitory factors such as blood, salts or lipids.

Previous transmission studies have evaluated the prion decontamination process of wire^25^,^26^,^31^,^32^,^33^,^34^. Among the inactivating methods for prion, we selected two commonly used inactivation methods, treatment with NaOH (1 mol/L NaOH for 2 h) or SDS solution (3% (w/v) SDS at 100 °C for 10 min), to see whether the wire-QuIC can be used for quantitative evaluation. Similar to bioassay studies^25^,^26^, the positive signals of wire-QuIC with prion seeds disappeared after treatment with NaOH. However, SDS treatment had no significant effect. A previous study reported that a mouse adapted human prion strain, Fukuoka-1, could be completely inactivated by boiling with 3% (w/v) SDS for at least 3 min^35^. Three reasons may explain this controversial result. (1) Seeding activity is not equal to infectivity. According to a previous report using 263K scrapie, the LD_50_ of the hamster brain was approximately 10–50-fold lower than the SD_50_^28^. (2) One hundred degrees Celsius is not enough for SDS inactivation, and “boiling” is an important factor. We cannot exclude this possibility without direct evaluation of these two conditions. (3) Each prion strain has a different sensitivity against SDS. We used hamster 263K or human sCJD for evaluation instead of the mouse-adapted Fukuoka-1 strain. Lemmer *et al.* also indicated that 5% (w/v) SDS treatment at 90 °C could not inactivate 263K-prion^26^. Other groups also showed that rodent adapted prions have a different sensitivity to SDS compared with naturally developed original prions^36^.

We also tested a new washing procedure designed for endoscopes using wire-QuIC. This new procedure washes the objects with electrolysed water in combination with sonication to remove organic substances and to inactivate microorganisms and viruses (unpublished). As shown in Fig. 6, this washing procedure can decontaminate prion pathogens completely from wire. High concentrations of sodium hydroxide, sodium hypochlorite or prolonged steam sterilisation are known to be effective against prion. However, some instruments, such as flexible endoscopes, cannot withstand the heat and high concentrations of disinfectants, resulting in the discarding of instruments after use in patients with CJD^17^. Therefore, this new washing procedure will reduce the risk of accidental transmission of prion. However, experiments need to be substantiated by transmission experimental data because the negative RT-QuIC reaction does not necessarily exclude the presence of infectivity on the instrument.

Taken together, the present study indicates that wire-QuIC is a useful method to evaluate washing procedures for prion contamination; however, further studies are needed in order to determine the quantitative relationship between QuIC positivity and infectivity of human prions.

## Methods

### Recombinant prion protein

Recombinant prion protein (recPrP) from Syrian hamster (recShaPrP23-231) or human (recHuPrP23-231) construct were expressed in *Escherichia coli* strain BL21 (DE3) (Stratagene, La Jolla, CA, USA) and purified as previously described^29^. Concentrations of recPrPs were determined by measuring the absorbance at 280 nm. After purification, aliquots of proteins were stored at −80 °C in distilled water.

### Preparation of brain tissue

For hamster prion (263K), brain tissues from Syrian golden hamsters infected with scrapie strain 263K were collected following euthanisation at the clinical stage of disease. Animal care and experimental procedures were performed in accordance with the Regulations and Guidelines for Animal Experimentation of Nagasaki University, reviewed by the Institutional Animal Care and Use Committee of Nagasaki University and approved by the president of Nagasaki University (ID: 1107040937). For human prion, brain tissue from a human prion disease (sCJD) patient was obtained for use in this study. Written informed consent to participate in the study was given by the patient's family. The protocol for investigation was approved by the Ethics Committee of Nagasaki University Hospital (ID: 10042823), and the study was registered with the University Hospital Medical Information Network (ID: UMIN000003301). The methods were carried out in accordance with the approved guidelines. BH in phosphate-buffered saline (PBS) were prepared (10% w/v) using a multi-bead shocker (Yasui Kikai, Osaka, Japan). After centrifugation at 2,000 g for 2 min, supernatants were collected and stored at −80 °C. Dilutions of BH were carried out in PBS immediately prior to the reactions. For Wire-QuIC reaction, stainless steel wires (SUS304, RKC Instrument Inc., Kanagawa, Japan; diameter 0.2 mm) were cut into 5-mm-long pieces. In order to contaminate wires with BH *in vitro*, wires were incubated with several concentrations of BH and air-dried at room temperature for 1 day in a Petri dish.

### Real-time quaking-induced conversion reaction (RT-QuIC)

RT-QuIC was performed as previously described^30^. Briefly, 95 μL of reaction buffer (50 mM PIPES pH 7.0, 500 mM NaCl, 1 mM EDTA and 10 μM Thioflavin T (ThT) including 80 μg/mL of recHamPrP23-231 for 263K-BH, or 100 μg/mL of recHuPrP23-231 for sCJD-BH) were loaded into wells of a 96-well optical-bottom black plate (Thermo Fisher Scientific 265301, MA, USA). Diluted BH (5 μL) was used for seeding. For the wire-QuIC reaction, air-dried wire was used. Ninety-six-well plates were covered with sealing tape (Greiner bio-one 676060, Frickenhausen, Germany) and incubated at 37 °C in a plate reader (Infinite F200 PRO fluorescence plate reader; Tecan, Zurich, Switzerland) with intermittent shaking, consisting of shaking (432 rpm orbital) for 30 sec and no shaking for 30 sec, with a 2-min pause to measure the fluorescence. ThT fluorescence measurements were taken every 10 min at 440 nm excitation and 485 nm emission wavelengths. Four replicates of each diluted sample were measured. Each curve represents a single well.

### Calculation of seeding dose

Seeding dose 50% (SD_50_), analogous to a bioassay’s lethal dose 50% (LD_50_), were calculated using the amount of BHs which cause RT-QUIC positive signal of 50% of the wells^37^.

### Decontaminations

Air-dried wires with attached BH were incubated for decontamination in 1 mol/L of NaOH solution for 2 h or 3% (w/v) Sodium dodecyl sulfate (SDS) solution at 100 °C for 10 min. Subsequently, wires were rinsed three times in distilled water for 1 min, and were air-dried again.

The new washing procedure for rigid endoscopes, for which electrolysis water and sonication were used, in collaboration with Kripton Co., Ltd. and Kyowakiden Industry Co., Ltd. in the project of “Program to support development of medical equipment and devices to solve unmet medical needs 2012, 2013” and “Development of Medical Device through Collaboration between Medicine and Industry 2014” under the Ministry of Economy, Trade and Industry (METI), Japan, was performed. To prevent diffusion of the pathogen, the same washing process was performed in 1.5 mL tubes. Electrolysed alkaline and acidic water were prepared in the electrolysis apparatus. The apparatus consists of anode and cathode plates, made of titanium and coated with platinum, that are separated by an electrolytic diaphragm (Y-9201T, Yuasa Membrane Systems Co. Ltd., Tokyo, Japan). The electrolysed water were collected in 500 mL bottles and used for the experiment. Our new washing procedure consisted of five processes to perform disinfection. Wires that attached BH were kept separately from each other in 1.5 mL tube, and were then pre-washed in tap water. Wires were then treated with electrolysed alkaline water while being sonicated at 45 kHz. Subsequently, wires were rinsed in water with sonication, and treated with electrolysed acidic water, followed by rinsing in tap water. Alkaline treatment and acidic water processing were performed sequentially for 3 min each.

## Additional Information

**How to cite this article**: Mori, T. *et al.* A direct assessment of human prion adhered to steel wire using real-time quaking-induced conversion. *Sci. Rep.* **6**, 24993; doi: 10.1038/srep24993 (2016).

## Supplementary Material

Supplementary Information
