# Supplementary material for: A direct assessment of human prion adhered to steel wire using real-time quaking-induced conversion
Source: Sci Rep. 2016 Apr 26;6:24993. doi: 10.1038/srep24993 (PMC4845018; doi:10.1038/srep24993)
Supplement: Supplementary Information [file srep24993-s1.pdf]

Supplementary Information

A direct assessment of human prion adhered to steel wire using real-time quaking-induced conversion

Tsuyoshi Mori[1]*, Ryuichiro Atarashi[1], Kana Furukawa[1], Hanae Takatsuki[1], Katsuya Satoh[2], Kazunori Sano[3], Takehiro Nakagaki[1], Daisuke Ishibashi[1], Kazuko Ichimiya[4], Masahisa Hamada[4], Takehisa Nakayama[4] and Noriyuki Nishida[1]

[1]Department of Molecular Microbiology and Immunology, Nagasaki University Graduate School of Biomedical Sciences, 1-12-4 Sakamoto, Nagasaki 852-8523, Japan

[2]Departmenrt of Locomotive Rehabilitation Science, Nagasaki University Graduate School of Biomedical Sciences, 1-7-1 Sakamoto, Nagasaki 852-8523, Japan

[3]Department of Physiology and Pharmacology, Faculty of Pharmaceutical Sciences, Fukuoka University, 8-19-1 Nanakuma, Jonan-ku, Fukuoka 814-0180, Japan

[4]Kripton Co., Ltd., Dai 12 Daitetsu Bldg. 7F. 4-3-12 Yotsuya, Shinjuku-ku, Tokyo 160-0004, Japan

Supplementary Figure S1

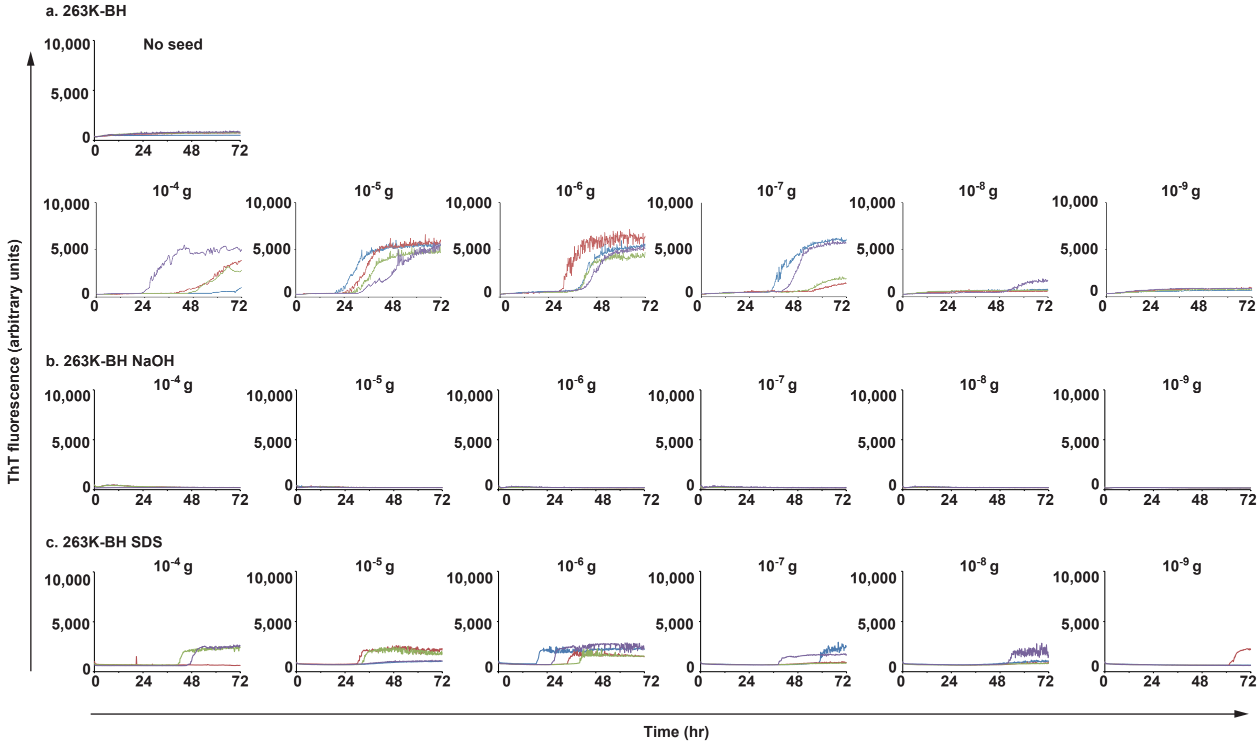

Inactivation of 263K hamster prion seeds treated with NaOH or SDS solution, as evaluated by RT-QuIC reactions.

Dilutions of 263K-BH (10-fold) were treated with 1 mol/L of NaOH solution for 2 hr (b) or 3% (w/v) SDS solution at 100°C for 10 min (c). RT-QuIC reactions were performed to measure the residual prion-seeding activities.
